# Ultra-Hypofractionated Re-Irradiation with Anti-PD-1 Immunotherapy for Locoregionally Recurrent (after Radical Chemo-Radiotherapy) Non-Small Cell Lung Cancer

**DOI:** 10.3390/cancers15205083

**Published:** 2023-10-20

**Authors:** Konstantinos Filippatos, Ioannis M. Koukourakis, Stavros Anevlavis, Axiotis Giaktzidis, Michael I. Koukourakis

**Affiliations:** 1 Department of Radiotherapy-Oncology, Medical School, Democritus University of Thrace, 68100 Alexandroupolis, Greece; filippkostas@gmail.com (K.F.); axiotakis@gmail.com (A.G.); 2 Radiation Oncology Unit, 1st Department of Radiology, “Aretaieion” University Hospital, Medical School, National and Kapodistrian University of Athens (NKUOA), 11528 Athens, Greece; koukourioannis@gmail.com; 3 Department of Pneumonology, Medical School, Democritus University of Thrace, 68100 Alexandroupolis, Greece; sanevlav@med.duth.gr

**Keywords:** non-small-cell lung cancer 1, ultra-hypofractionated radiotherapy, nivolumab, pembrolizumab, re-irradiation, recurrent tumor

## Abstract

**Simple Summary:**

Concurrent administration of anti-PD-1 immunotherapy with one or two fractions of ultra-hypofractionated (8 Gy/fraction) radiotherapy for patients with locoregionally recurrent non-small cell lung cancer (NSCLC) after radical chemo-radiotherapy was examined in a cohort of 11 patients. This immuno-radiotherapy scheme was safe and provided 81.8% objective response rates. The complete response rate was 27.2%, while tumor regression by 80–100% of initial dimensions was noted in 63.5% of patients. The 22-month locoregional relapse-free rate was 54.5%, while the projected 2-year disease-specific overall survival was 62%. These encouraging results provide the basis to pursue immuno-radiotherapy trials with ultra-hypofractionated radiotherapy schemes in this ill-fated group of NSCLC patients.

**Abstract:**

Large fractions of radiotherapy of 8 Gy (ultra-hypofractionated RT, ultra-hypoRT) promote anti-tumor immune responses that have been clinically substantiated in combination trials with immune checkpoint inhibitors (ICIs). In the current study, we postulated that ultra-hypoRT in combination with ICIs may enhance tumor clearance in NSCLC patients with locoregional relapse after radical chemo-RT. Between 2019 and 2021, eleven patients received re-irradiation with one or two fractions of 8 Gy concurrently with anti-PD1 immunotherapy (nivolumab or pembrolizumab). RT-related toxicities were negligible, while immune-related adverse events enforced immunotherapy interruption in 36% of patients. The overall response rate was 81.8%. Tumor reduction between 80 and 100% was noted in 63.5% of patients. Within a median follow-up of 22 months, the locoregional relapse-free rate was 54.5%, while the projected 2-year disease-specific overall survival was 62%. The results were independent of PD-L1 status. The current report provides encouraging evidence that a relatively low biological dose of RT delivered with 8 Gy fractions is feasible and can be safely combined with anti-PD-1 immunotherapy. Despite the low number of patients, the significant tumor regression achieved and the long-lasting locoregional control and overall progression-free intervals provide a basis to pursue immuno-RT trials with U-hypoRT schemes in this group of NSCLC patients of poor prognosis.

## 1. Introduction

Inoperable for medical or surgical reasons, stage III non-small cell lung cancer (NSCLC) is a common human malignancy, most frequently treated with concurrent cisplatin-based chemo-radiotherapy (chemo-RT) [1]. Patients who respond to chemo-RT further benefit from consolidation immunotherapy with the anti-PD1 durvalumab monoclonal antibody. Mature analysis of the PACFIC trial showed an estimated 42.9% 5-year survival in the immunotherapy group vs. 33.1% in the chemo-RT group [2]. However, about thirty percent of these patients present with locoregional recurrence during their follow-up [3].

Re-irradiation of locoregionally recurrent disease is a widely applied therapeutic option [3], although no randomized trials test this approach against chemotherapy or immunotherapy alone. Conventional RT with 3D or IMRT/VMAT techniques have been applied, delivering doses up to 60 Gy, reporting a median overall survival (OS) of 7–13 months and a 2-year locoregional control of 40% [4,5,6]. Stereotactic ablative RT (SBRT), delivering high doses per fraction, has also been applied in several studies providing an OS of 12–40 months and 2-year local control rates of 54% [7,8,9,10,11]. The National Comprehensive Cancer Network guidelines for recurrent NSCLC suggest external beam RT (standard or ablative) for resectable recurrences, concurrent chemo-RT for nodal recurrences, and palliative RT for superior vena cava syndrome or hemoptysis (https://www.nccn.org/professionals/physician_gls/pdf/nscl.pdf; last accessed on 7 October 2023). Surgery, endobroncheal laser, or photodynamic therapy is also suggested for selected patients. 

Activation of the immune system to promote immune tumor rejection by primed T-cells and monocytes has been established as a powerful therapeutic approach after the introduction of immune checkpoint inhibitors in the oncology clinical practice. Anti-PD1 and anti-PD-L1 immunotherapy have been rapidly adopted as first-line therapy, either as monotherapy or combined with chemotherapy, for patients with metastatic NSCLC [12]. Indeed, pembrolizumab provided objective responses in 26–39% of patients and a 5-year survival of 20% in PD-L1-expressing cases [13]. Nivolumab in combination with the ipilimumab anti-CTLA4 antibody provided a 15.6-month median survival in stage IV disease vs. 10.9 months when patients were treated with chemotherapy alone, and this benefit was independent of tumor PD-L1 expression status [14]. 

Strong experimental evidence suggests that tumor irradiation, especially when large RT fractions are applied, produces a cascade of molecular changes that render cancer cells more vulnerable to anti-tumor immunity [15]. This ‘radio-vaccination’ effect further stimulates anti-tumor immune responses against cancer cells outside the radiation portals, boosting radiation’s so-called ‘abscopal’ effects [16]. Fractions of 8 Gy (ultra-hypofractionated RT, ultra-hypoRT) seem to have a stronger effect in inducing the IFN-type-I response than standard fractionation or larger RT fractions [17]. It has been, therefore, postulated that the combination of immune checkpoint inhibitors (ICIs) with 8 Gy RT fractions is the optimal way to therapeutically exploit the efficacy of RT to empower the anti-tumor effects of immunotherapy at the ‘abscopal’ level. Nevertheless, such synergy is also expected to produce ‘in-field’ immune effects that facilitate tumor eradication by combining the killing effects of RT with cancer cell clearance by activated lymphocytes and monocytes. 

We hypothesized that combining anti-PD-1 immunotherapy with ultra-hypoRT re-irradiation would improve the outcomes of patients with locoregionally recurrent NSCLC pretreated with radical chemo-RT. To our knowledge, this is the first study in the literature reporting results from an analysis of a small series of patients with recurrent NSCLC treated with ICIs (pembrolizumab or nivolumab) concurrently with one or two fractions of 8 Gy of RT.

## 2. Materials and Methods

Eleven patients with stage III NSCLC cancer who recurred locoregionally after concurrent radical chemo-RT with cisplatin (50 mg/m^2^ every two weeks) and Nab-Paclitaxel (150 mg/m^2^ every two weeks) between 2015 and 2021 were retrospectively analyzed. These patients had not received durvalumab maintenance immunotherapy, as at the time of patient therapy (2015–2021), this had not been a standard treatment (the European Medicines Agency approved the regimen in December 2020). The RT dose delivered to the primary tumor was 49 Gy using an accelerated hypofractionated scheme, delivering 3.5 Gy/fraction for 14 fractions. This RT schedule, applying a simultaneous integrated dose technique (2.7 Gy/fraction to the nodal areas and 3.5 Gy/fraction to the primary tumor area), has been extensively tested for safety and efficacy by our group in combination with chemotherapy and is widely applied in our department [18,19,20]. This delivers an equivalent dose delivered in 2 Gy fractions (EQD2 or normalized total dose (NTD)), calculated for a lung α/β-ratio of 3 Gy, of 63.7 Gy within 18 days [21]. Considering an acceleration of 26 days (as a conventionally fractionated RT schedule that delivers 64 Gy demands 44 days for its completion) and a λ-value of 0.2 Gy/day (dose estimated to be daily consumed on cancer cell repopulation during RT), the estimated EQD2 with time correction (EQD2-T) is 68.9 Gy.

In the current study, patients who recurred after the above chemo-RT scheme received anti-PD1 immunotherapy combined with re-irradiation (immuno-RT) of the recurrent disease, using 8 Gy ultra-hypoRT. Patients were recruited in the context of an ongoing research protocol approved by the Ethics and Research Committee of the University Hospital of Alexandroupolis (Θ87/ΔΣ15/13-11-2019). This protocol studies the role of anti-PD1 immunotherapy in patients with recurrent carcinomas of various origins after RT and cisplatin-based chemotherapy. Palliative RT with large fractions (e.g., 8 Gy), as suggested by the dose fractionation guidelines of the UK Royal College of Radiologists, was allowed at the discretion of oncologists (https://www.rcr.ac.uk/publication/radiotherapy-dose-fractionation-third-edition; last accessed on 7 October 2023). 

Recurrence confirmation was based on radiological assessment and PET-CT imaging, and no biopsy had been performed. Patient and disease characteristics are reported in Table 1. The time of recurrence since the end of the previous chemo-RT ranged from 6 to 60 months (a median of 13 months). Three patients had additional sites of recurrence outside the radiation portals, as shown in Table 1. The follow-up of patients after re-irradiation ranged from 4 to 40 months (a median of 22). 

### 2.1. Pretreatment and Treatment Evaluation

Diagnosis of disease recurrence was confirmed through CT-scan, MRI, and/or PET-CT imaging. Full blood counts, ECG, glucose levels, and biochemical kidney and liver function were assessed before therapy to provide a baseline for monitoring the adverse events of immunotherapy (irAEs). Thyroid function (TSH, T3, and T4), C-reactive protein (CRP), and creatin phosphokinase (CPK) levels were also examined.

Response to therapy was assessed using CT or MRI scans performed 4 months after re-irradiation. The RECIST 1.1 criteria were used to define response [22]. Complete response (CR) refers to the elimination of detectable disease or documentation of a remnant scar measuring <5% of the initial dimensions. A decrease in the sum of the longest diameters (of all irradiated lesions) by more than 30% was considered a partial response (PR). Progressive disease (PgD) was defined as a >20% increase in the longest dimension. All other cases were considered as stable disease (SD). 

The NIH/NCI (National Institute of Health/National Cancer Institute) Common Terminology Criteria for Adverse Events (CTCAE) v 5.0 scale was used to score irAEs and RT-related acute toxicity [23]. The LENT-SOMA toxicity scale was used to score late radiation sequel [24]. 

### 2.2. Radiotherapy Details 

Treatment characteristics are reported in Table 2. RT was delivered using a volumetric modulated arc image-guided RT VMAT/IGRT technique, with a cone-beam CT performed before each RT fraction. GTV comprised all radiologically detectable disease, and a margin of 0.5 cm was considered for CTV. A margin of 0.5 cm beyond CTV, followed by manual adjustment, was applied for PTV. Locoregionally recurrent disease was treated with 1 fraction of 8 Gy, while in three patients, 1 additional fraction of 8 Gy was administered after a new simulation and RT planning. Organs at risk include the lungs, spinal cord, and esophagus. Metastatic lesions (disease outside the pre-irradiated areas) received three weekly fractions of 8 Gy using a VMAT/IGRT technique. 

The EQD2 delivered at 1 and 2 fractions of 8 Gy, calculated for lung α/β-ratio of 3 Gy (for normal lung tissue), was 17.6 Gy and 35.2 Gy, respectively. 

### 2.3. Immunotherapy 

All patients received IV anti-PD-1 immunotherapy starting on the day of the administration of RT. Six patients received pembrolizumab, at a dose of 200 mg every 3 weeks. Five patients received nivolumab at a dose of 240 mg IV every two weeks. Immunotherapy continued until the documentation of disease progression or after documentation of potentially severe irAE, excluding thyroid dysfunction that was successfully treated with hormone replacement. For patients who achieved CR, immunotherapy continued for 24 months. Six patients had positive expression of PD-L1 in immunohistochemical analysis, and five received pembrolizumab. The rest received nivolumab immunotherapy.

### 2.4. Statistical Analysis

The endpoints of the current study were RT tolerance, evaluation of irAEs, tumor response rates, locoregional progression-free survival (LPFS), and disease-specific OS. Kaplan–Meier survival curves and waterfall graphs were plotted using the GraphPad Prism version 7.0 program. 

## 3. Results

### 3.1. Radiotherapy Toxicity

RT was well tolerated without any acute RT-related side effects from the esophagus, lung, trachea, or skin. Regarding late radiation toxicity, five patients exhibited marginally detectable radiological deterioration of the pre-existing in-field lung fibrosis, albeit without any clinical symptoms. Table 3 reports the individual RT-related toxicities.

### 3.2. Immunotherapy Adverse Events

Table 3 reports the individual immunotherapy-related adverse events. One patient interrupted immunotherapy at 4 months because of grade 3 renal dysfunction, as indicated by creatinine rise (9%), which regressed under treatment with oral methylprednisolone (16 mg daily for one month). Two patients developed grade 2 fatigue (18%) at 8 and 12 months, respectively, after the onset of immunotherapy, without CPK elevation (indicating absence of rhabdomyolysis), and were treated with a similar methylprednisolone schedule. Symptoms regressed within two months. One additional patient developed a generalized skin rash (9%) 7 months after the onset of treatment. The patient was treated with oral methylprednisolone, and symptomatology regressed within 3 months. Thus, 4/11 (36%) patients interrupted immunotherapy because of irAEs. None of these irAEs were life-threatening. One additional patient developed hypothyroidism (9%) that was well controlled with thyroid hormone replacement without interruption of immunotherapy.

### 3.3. Tumor Response

A CT scan performed 4 months after irradiation confirmed CR in 3/11 (27.2%) of patients, while an almost CR, thus a PR with 80–90% of reduction of tumor dimensions, was noted in an additional 4/11 (36.3%) of patients. Two more patients (18.1%) achieved PR (30–60% of tumor dimension reduction). One patient had disease stabilization, while one patient progressed during therapy. The overall response rate (CR + PR) was 81.8%. All three metastatic sites treated with three fractions of ultra-hypoRT regressed completely.

**Figure 1:**
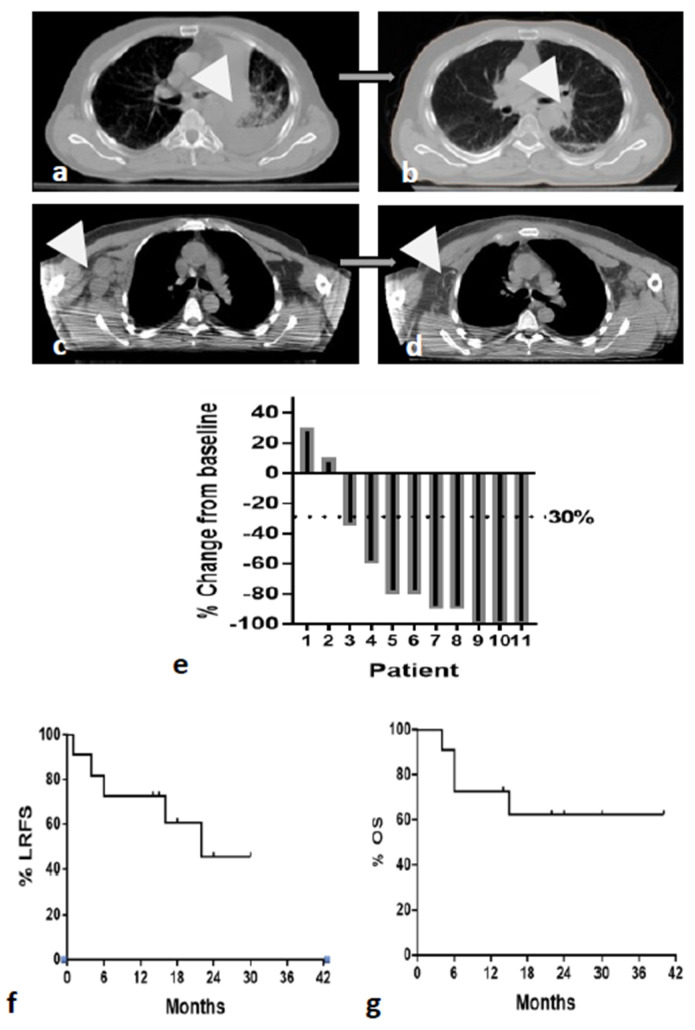
Re-irradiation of NSCLC patients with ultra-hypofractionated immuno-RT: (**a**–**d**) typical images of complete responses (white arrows) in two patients ((**a**,**c**) before RT; (**b**,**d**) after RT); (**e**) waterfall plot analysis of local lung tumor dimension changes assessed 4 months after RT; (**f**) locoregional relapse-free (LRFS) Kaplan–Meier survival curves; (**g**) disease-specific overall survival (OS) Kaplan–Meier survival curves.

Typical CT images before and after immuno-RT are shown in Figure 1a–d. A waterfall plot of changes in lung tumor dimensions from baseline is shown in Figure 1e. There was no association between tumor response and PD-L1 status.

### 3.4. Survival Analysis

Within a median follow-up of 22 months, 6/11 patients were alive. Four deaths from disease and one death from other causes were recorded. The median survival was undefined, while the projected 2-year survival was estimated to 62% (standard error of 15%). The range of time to disease-specific death or last follow-up of patients alive was 4–40 months. Five of eleven patients relapsed in the RT fields, while none of the metastatic lesions treated with ultra-hypoRT showed any signs of local disease progression. The median LRFS was 22 months, while the projected 2-year LRFS rate was 45% (standard error of 17%). The time range to locoregional relapse or last follow-up of patients without recurrence was 4–40 months. Kaplan–Meier LRFS and OS are shown in Figure 1f,g.

Table 4 reports the patient individual data and treatment outcome.

## 4. Discussion

Changes in the cancer cell phenotype and tumor microenvironment induced by radiation unmask cancer’s foreign nature and facilitate tumor stroma infiltration by educated cytotoxic T-cells and monocytes [15]. Tumor irradiation with one to three fractions of 8 Gy results in double-stranded DNA fragments in the cytoplasm of cancer cells and induction of the cGAS/STING (cyclic GMP-AMP synthase and stimulator of interferon genes) pathway [17]. RT also induces the type-I IFN pathway through STING-independent mechanisms that activate the transcription of many IFN-regulated genes [25,26]. Secretion of type-I IFN proteins activates dendritic cells, T-cells, and monocytes residing in the tumor stroma that drive anti-tumor immune responses. 

In addition, RT induces multiple pathways related to immune response activation. An important pathway is the activation of HLA-class-I expression that enhances the presentation of foreign peptides to dendritic cells that travel to the regional tumor-draining lymph nodes to activate T-cells against cancer-specific antigens [27]. T-cells will thereafter travel through the bloodstream to attack metastatic cancer foci and produce the so-called ‘abscopal’ (out of RT field) effects of RT but also to attack the already irradiated primary tumor to promote ‘in-field’ cancer cell eradication of remnant cancer cells that survive during and after roRT [28]. Furthermore, the interactions of RT with the tumor stroma to elicit chemotactic stimuli that attract peripheral lymphocytes and macrophages and facilitate endothelial transmigration into the tumor microenvironment further support the importance of RT in empowering the efficacy of immunotherapy in ‘in RT-field’ and ‘out of RT-field’ tumor areas [29,30].

The effects of RT as an enhancer of the activity of ICIs have been exploited in recent clinical research in metastatic NSCLC. Specifically, an analysis of two phase 2 randomized trials (PEMBRO-RT and MDACC) compared the efficacy of pembrolizumab alone vs. pembrolizumab with either 3 fractions of 8 Gy-ultra-hypoRT or 3 Gy/fraction-hypofractionated RT (15 fractions) directed to multiple metastatic sites, sparing at least one measurable metastatic lesion without irradiation to use it as a control for abscopal effect manifestation. The study suggested that irradiation improved the median OS from 8.7 to 19.2 months compared to immunotherapy alone and, furthermore, increased the abscopal response rates from 19.7% to 41.7% and the abscopal control rate from 43.4% to 65.3% [31]. Results of other interestingly designed trials await the confirmation of the efficacy of metastatic lesion irradiation in triggering abscopal effects during immunotherapy for NSCLC [32,33]. 

In terms of potentiation of immunotherapy to eradicate persistent irradiated NSCLC, the PACIFIC trial showed impressive results of durvalumab administered after the completion of radical chemo-RT in stage III patients. A significant reduction of disease progression and prolongation of OS was confirmed (estimated 5-year survival rates of 42.9% vs. 33.1% for durvalumab vs. placebo arms, respectively) [2]. A recent randomized trial focused on the role of immunotherapy in eradicating early-stage I–II NSCLC treated with concurrent SBRT, demonstrating that nivolumab improved the 4-year event-free survival from 53% to 77% [34]. This encouraging study provides the basis for pursuing immuno-RT trials to replace surgery with less invasive therapeutic approaches in patients with early operable NSCLC.

As immunotherapy’s ‘in-field’ effects are gradually being documented, the role of ICI combination with RT in patients with recurrent localized disease after previous chemo-RT becomes a promising area for clinical research. Since large RT fractions have, at least experimentally, shown a more portent efficacy to induce anti-tumor immune responses, fractions of 8 Gy (ultra-hypoRT) or ablative approaches (fractions of 12–25 Gy) delivered with modern VMAT and stereotactic techniques have become appealing for the re-treatment of loco-regionally relapsed disease. For now, there is a lack of clinical studies published in the literature. In a recent study, we showed that re-irradiation of HNC recurrent tumors with 8 Gy fractions together with anti-PD-1 immunotherapy provides high and long-lasting control rates above the expected from chemotherapy or re-irradiation [35]. 

The current study reports encouraging results in a small series of NSCLC patients with locoregionally recurrent NSCLC, treated with one or two fractions of localized 8 Gy RT and anti-PD-1 immunotherapy. The biological dose delivered, equivalent to 17.6 Gy and 35.2 Gy, is considered low to anticipate high tumor control rates. Indeed, re-irradiation studies (referenced below) consider doses above 60 Gy, which, although expected to offer measurable and long-lasting tumor control, may eventually result in severe lung fibrosis and other RT-related late side effects. Given the high biological dose of 69 Gy delivered to our patients at first irradiation and the rather short 13-month median interval of recurrence documentation in this series of patients, this low re-irradiation dose was chosen to avoid excessive incidence of lung fibrosis, anticipating the treatment benefit from the potentiation of immunotherapy. Indeed, symptomatic pneumonia or lung fibrosis incidence was negligible in our patients. Radiological documentation of mild deterioration of pre-existing fibrotic changes was evident in 5/11 patients, while immune-related pneumonitis was not observed.

Nevertheless, other irAEs enforcing immunotherapy interruption were noted in 36% of patients, which regressed under methylprednisolone treatment within 1–2 months. None of the irAEs was life-threatening, and most patients received an efficient number of immunotherapy cycles before the manifestation of irAEs. The overall response rate was 81.8%, whereas 63.5% of patients had strong responses with tumor reduction between 80 and 100%. Within a median follow-up of 22 months, 6 out of 11 (54.5%) patients were without locoregional disease, while the projected 2-year disease-specific OS was 62%. The results were not affected by the PD-L1 status, but the study was too small to extract reliable conclusions.

The above results appear promising compared to published studies examining re-irradiation without immunotherapy to treat locoregionally recurrent after RT NSCLC. Considering the relatively low biological radiation dose delivered, the efficacy of immuno-RT with 8 Gy radiation fractions becomes intriguing. Most studies in the literature provided high doses for re-irradiation exceeding 60 Gy. In a recent study, Grambozov et al. reported a series of 31 NSCLC recurring locally after chemo-RT, delivering high radiation doses through different fractionation schedules ranging from hyperfractionated to conventional, hypofractionated, and ablative RT, where the median control interval was 7.9 months [36]. In a study by Yang et al., 50 patients received conventionally fractionated re-irradiation with a median RT dose of 51.1 Gy, and the median progression-free survival was only 5.9 months [37]. Similarly, poor results were reported in a series of 52 NSCLC patients treated with conventional or mildly hypofractionated RT, delivering a median overall dose of 38.5 Gy (ranging from 20 to 60 Gy), where the median local progression-free survival and OS were 6.5 and 9.2 months, respectively [38]. Nevertheless, other authors have observed better results. Hong et al. published on 31 NSCLC patients treated with high-dose conventional re-irradiation, where the median LRFS and OS were 15.4 and 20.4 months, respectively [39]. However, the median dose of re-irradiation was quite high (68.8 Gy). In another series of 26 patients treated with SBRT re-irradiation with a median of 61.2 Gy, the local control rate was as high as 80%, and the actuarial 2-year progression-free survival was 37% [40]. Lee et al. also reported a 63.3% 2-year local control rate after re-irradiation of recurrent NSCLC with 4–8 large RT fractions delivering a dose of 48–60 Gy [41].

An important limitation of the current report is the small number of patients and the study’s retrospective nature. Moreover, the lack of a matched control group is another limitation that should be considered. Overall, the results should be regarded as preliminary and carefully designed prospective phase I/II dose-escalation trials; increasing the number of 8 Gy fractions according to the pre-existing lung toxicity is necessary to extract safe conclusions. It is stressed that the site of relapse in the lungs (central or peripheral), the tumor volume and the unavoidable normal-lung volume included in the re-irradiation planning, the pre-re-irradiation dose levels delivered to the normal anatomical structures, the already established RT lung fibrosis, and the remnant respiratory reserves create a complex background that should be carefully taken into account when designing such prospective trials. Individualization of re-irradiation (irradiation area and dose) is inevitable.

## 5. Conclusions

The current report provides encouraging evidence that a low biological dose of RT delivered with one or two 8 Gy fractions is feasible and can be safely combined with anti-PD-1 immunotherapy to treat locoregionally recurrent NSCLC patients pretreated with radical chemo-RT. The dramatic tumor regression achieved in more than 80% of patients and the long-lasting locoregional control and overall progression-free intervals provide a basis to pursue immuno-RT trials in this group of NSCLC patients with poor prognosis. Escalation of the RT number of fractions can also be tested, given the low early and late toxicity rates noted. Furthermore, the value of immuno-RT with ultra-hypoRT or SBRT schedules for the radical therapy of non-metastatic NSCLC, recently confirmed in the early stages of the disease [34], should be further evaluated.

## Figures and Tables

**Table 1:** Patient and disease characteristics.

Number of Patients	11
Age	44–78 (median 71)
Gender	
Male	9
Female	2
Histology	
Squamous	9
Adenocarcinoma	1
Large cell undifferentiated	1
PD-L1 expression (*)	
Negative	5
Positive	6
Previous therapy	
Chemo-radiotherapy	11 (**)
Site of recurrence	
In RT field	11
Contralateral lung	1
Supraclavicular/axillary	1
Upper mediastinum	1

(*) initial pre-chemo-radiotherapy biopsy; (**) cisplatin/Nab-Paclitaxel + radiotherapy.

**Table 2:** Treatment characteristics.

	Number of Patients
Radiotherapy (RT) to locoregional	
previously irradiated sites	8
8 Gy × 1 fraction	3
8 Gy × 2 fractions	
RT to non-previously	
irradiated sites	
8 Gy × 3 fractions	3
Concurrent immunotherapy	
Nivolumab	5
Pembrolizumab	6

**Table 3:** Individual RT and immunotherapy-related toxicities.

Number of Patients	Irradiated Site	RT Lung Toxicity	RT Other Toxicity	irAEs	No IO Cycles/IO Type
1	lung	(***)	none	none	6/N
2	lung	none	none	skin rash	12/P
3	lung	none	none	none	28/P
4	lung	(***)	none	asthenia	20/P
5	lung	none	none	hypothyroidism	25/N
	nodes (*)	none	none	none	
6	lung	(***)	none	renal	8/P
7	lung	none	none	asthenia	15/N
8	lung	none	none	none	19/P
	metastasis (**)	none	none	none	
9	lung	(***)	none	none	6/N
10	lung	none	none	none	28/N
11	lung	(***)	none	none	15/P
	mediastinum	none	none	none	

(*) supraclavicular and axillary; (**) contralateral lung; (***) mild deterioration of pre-existing lung fibrosis. Abbreviations: iRAEs = immune-related adverse events; RT = radiotherapy; IO = immunotherapy; pt = patients; P = pembrolizumab; N = nivolumab.

**Table 4:** Individual patient, disease, and treatment characteristics, tumor response, local control, and survival.

Number of Patients	Age	Sex	Histo- logy	Grade	PD-L1 Status	Recurrence after CRT (Months)	Recurrence Site	Re-RT Dose (Gy/ Fractions)	IO	Response (% Change)	Local Control (Months) /Status	Survival (Months) /Status
1	77	f	S	2	−	12	lung	8/1	N	10	4/r	4/d
2	67	m	S	3	+	18	lung	8/1	P	−60	15	15/d
3	75	m	S	3	+	60	lung	8/2	P	−90	22	22
4	44	m	S	2	+	50	lung	8/1	P	−80	18	30
5	62	m	U	3	−	12	lung	8/1	N	−100	24	40
							nodes (*)	8/3		−100		
6	73	m	S	3	+	6	lung	8/1	P	−90	6/r	6/d
7	78	m	A	3	−	11	lung	8/2	N	−100	30	30
8	70	m	S	3	+	18	lung	8/1	P	35	16/r	30
							metastasis (**)	8/3		−100		
9	64	m	S	3	+	6	lung	8/2	N	−100	1/r	6/d
10	74	m	S	2	−	13	lung	8/1	N	−35	22/r	24
11	75	f	S	2	−	14	lung	8/1	P	−80	14	14
							mediastinum	8/3		−100		

(*) supraclavicular and axillary; (**) contralateral lung; Abbreviations: pt = patients; RT = radiotherapy; CRT = chemo-RT; m = male; f = female; S = squamous; A = adenocarcinoma; U = large cell undifferentiated; (−) negative; (+) positive; P = pembrolizumab; N = nivolumab; r = relapse; d = death.

## Data Availability

All data reported in the study are available in the files of patients kept in our Department.
